# Veterinarians and One Health in the Fight Against Zoonoses Such as COVID-19

**DOI:** 10.3389/fvets.2020.576262

**Published:** 2020-10-30

**Authors:** Roberta Torres de Melo, Daise Aparecida Rossi, Guilherme Paz Monteiro, Heriberto Fernandez

**Affiliations:** ^1^Faculty of Veterinary Medicine, Federal University of Uberlândia, Uberlândia, Brazil; ^2^Institute of Clinical Microbiology, Universidad Austral de Chile, Valdivia, Chile

**Keywords:** animal health, emerging zoonoses, forefront, infectious diseases control, public health

## Introduction

We are currently living in an unusual pandemic, due to the spread of Coronavirus Disease in 2019 (COVID-19), caused by the Severe Acute Respiratory Syndrome Coronavirus 2 (SARS-CoV-2). This virus forms a sister clade to the prototype human and bat severe acute respiratory syndrome coronaviruses (SARS-CoVs) (1). SARS-CoV-2 belongs to the *Coronavirinae* subfamily, family *Coronaviridae*. Virions are spherical, displaying club-shaped projections. Enveloped by a lipid bilayer envelope, SARs-CoV-2 has a positive-sense single-stranded RNA that encodes four structural proteins [proteins S (spike protein), E (envelope), M (membrane), and N (nucleocapsid)] and 16 non-structural proteins (2, 3).

The pathogen has spread around the world within a short period of time. In 8 months (from December 2019 to August 2020) it has reached more than 19 million cases with ~728 thousand deaths in more than 215 countries/territories/areas of the world. In addition to the challenges it poses to public health, it has provoked economic and social consequences due to the pandemic control measures, which are based on social distancing and the different quarantine concepts implemented throughout the world (4).

The pandemic originated in the city of Wuhan, in the south of China, where rapid economic growth promoted a high demand for animal protein and included regional eating habits, like consumption of exotic animals such as bats, snakes, and pangolins, which are normally traded in local food markets. Despite other possible theories, the precarious sanitary conditions and the lack of standards or biosafety models in these markets probably favored the transmission of infectious agents including viruses from the Corona group such as SARS-CoV-2, between animals and from animals to humans. The literature published to date indicates that the virus went through molecular changes due to the way that this pathogen propagates, leaving footprints (traces) in its genome, which can be identified (tracked) using phylogenetic analysis methods. This has allowed for the reconstruction of a model on the evolutionary history of the new coronavirus, showing that the strain that gave rise to SARS-CoV-2 has been circulating unnoticed in bats for 30–70 years and possibly adapted to pangolins, a species that harbors CoVs, sharing significant genomic homology with SARS-CoV-2 and showing extensive contact with humans in the Chinese markets (5–9).

The emergence and spread of zoonotic diseases like COVID-19 caused by SARS-CoV-2, indicates that veterinarians inhabit a central and primary position in the prevention of new and dangerous zoonoses that may impact human health. This is particularly true as this pandemic fits into the One Health concept, which considers the interactions between humans, animals, and the environment, and recognizes that human health is closely related to animal and environmental health (7). It is in these interfaces that veterinarians can play a relevant role in the prevention and detection of new zoonoses and determine which ones deserve at least some consideration.

## The Context of Emerging Zoonoses

It has been proven that at least 75% of emerging diseases have a zoonotic origin, having diverse animal species as their primary reservoirs. Striking examples of these zoonoses include epidemics and/or pandemics such as the Spanish flu (10), H1N1 (11), SARS, (12), MERS (13), and Ebola (14). All the etiological agents involved in those cases were originally found in animals and spread in humans. When a pathogen crosses species barrier, in most cases, the disease may not sustain or establish in the heterologous host. However, occasionally, there is a potential risk that it becomes more pathogenic and virulent, with consequences almost impossible to predict, as was experienced with the HIV virus (15).

The number of potential human lives lost as well as the high morbidity and imminent risks of epidemics/pandemics converging to result in the emergence of new diseases makes it imperative and necessary to intensify studies, in which veterinarians have the responsibility to identify and reveal the risks, critical points and other epidemiological aspects involved in the transmission of an infectious agent from the animal, environment, and human interfaces.

## Inclusion of The Veterinarian Professional

Considering the possible origin of the current pandemic, the intensification of studies in regions associated with high human activity alone, and with intense contact with wildlife, should become a priority in the prevention of emerging diseases, as such hotspots are decisive in the emergence of new epidemics and pandemics. There are several regions in the world in which it is possible to identify the presence of these hotspots, including low and middle-income countries in South America and Africa. Therefore, potential new pandemics such as COVID-19 pose serious health threats in underdeveloped countries where these hotspots occur. This type of hotspot can also be seen in developed countries especially in places where there is a greater vulnerability in poor populations (16). Therefore, the consequences of contact between wildlife and human activities can have consequences in rich countries.

It is necessary to reunite the forces and capacities of all of the involved actors and entities to avoid chaos and lack of control in the case of new emerging zoonotic diseases. The knowledge obtained through monitoring eventual ecological/epidemiological changes and the experience of previous studies in all knowledge areas will be effective tools to predict, prevent, and anticipate outbreaks by impacting zoonotic diseases such as COVID-19. This summarizes the veterinarian's role in public health, framed in the One Health concept.

Emerging and reemerging pathogens present challenges for public health systems worldwide. When considering animal interaction, the complexity of these challenges becomes even more evident. Despite the integration of the OIE and the WHO in the context of the One Health concept in 1960, the idea is still poorly explored in underdeveloped countries. Investment in studies that prioritize the investigation of the infectious agents present in wild animals associated with hotspots does not represent, at this moment a priority strategy for the prevention of pandemics by public health agencies. The implementation of this type of action would allow for the early identification of potential pathogens and the development of actions that could block and reduce opportunities for the pathogens to circulate freely and repeatedly among primary hosts and become highly infectious to humans.

## Some Specific Actions

**Figure 1:**
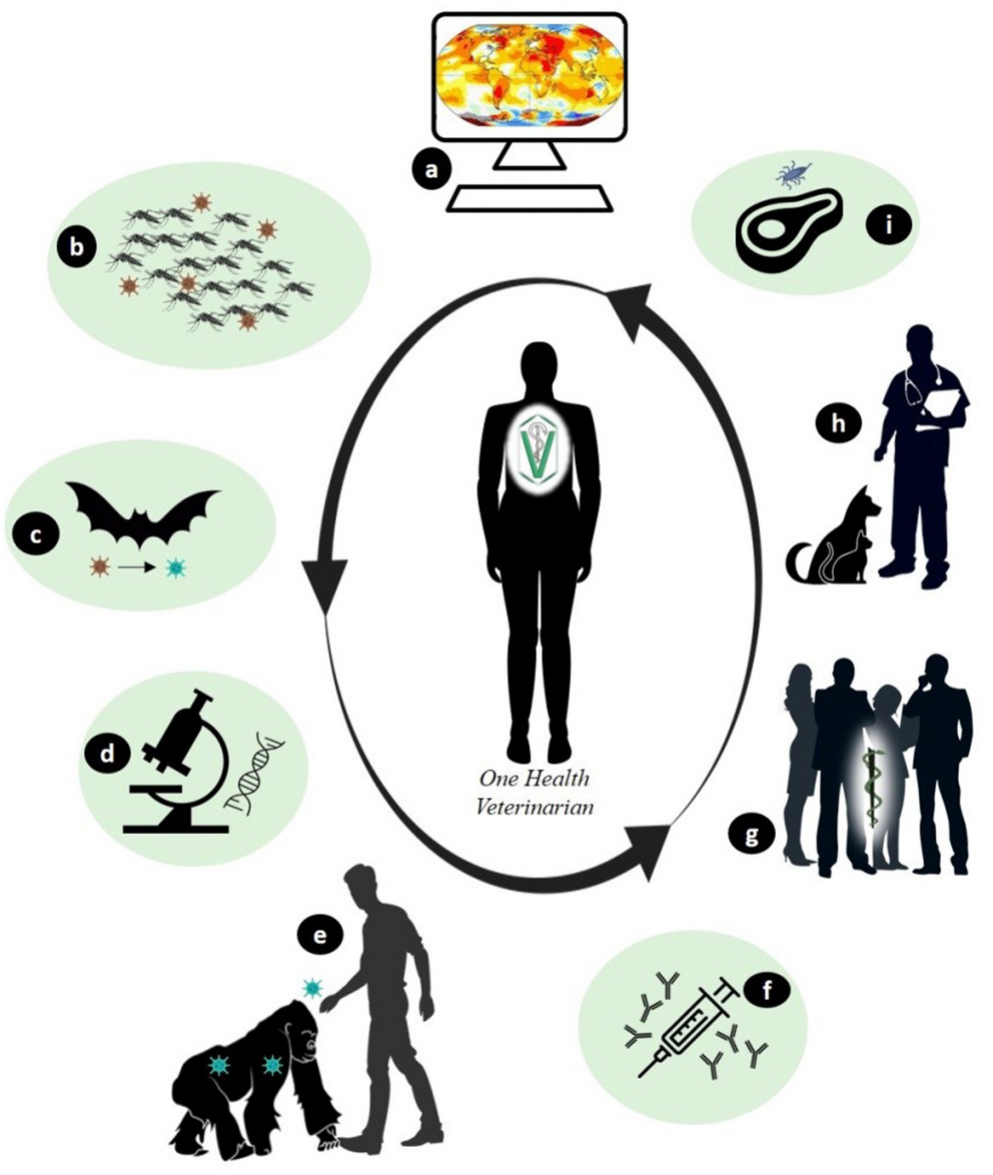
Examples of specific actions by the veterinarians to combat the emergence of zoonotic diseases. **(A)** - Studies to monitor multiple factors (environmental, temporal, and others) available in Geographic Information Systems that allow indicating changes for the modeling and forecasting of diseases. **(B)** - Environmental control that indirectly interferes in the control of vectors and/or hosts amplifying agents that transmit zoonotic diseases. **(C)** - Monitoring of wildlife through research that evaluates changes in the host and potential pathogens. **(D)** - *In vitro* investigation regarding the evolution of the characteristics of infectious agents over time. **(E)**-Health education through clarification to the population about the risks and care to be taken in human-animal contact. **(F)** - Production of diagnostic tests and vaccines using animal models based on comparative medicine. **(G)** - Application of translational medicine and zoobiquity in the exchange of experiences between teams of multi health professionals. **(H)** - Sanitary control in pets and production animals. **(I)** - Inspection and control of food of animal origin.

The chaos in the health services and consequently, in economic systems worldwide due to COVID-19 was at times the result of disobedience and non-compliance with the guidelines of preventive medicine, which outline the best strategies for containing the spread of the disease. Despite the knowledge and experience gained in previous epidemics, most governmental institutions are still not convinced of the veterinarian's role in this context. Veterinarians have experience in successfully managing outbreaks of diseases, such as brucellosis, tuberculosis, anthrax, foot-and-mouth disease, and rabies, in addition to controlling zoonotic pathogens in foods of animal origin (2, 17–20). Control measures, when strictly applied to animals, have resulted in a significant reduction of zoonoses in humans. Some of the specific actions of the veterinary profession are shown in Figure 1.

Practices such as zoobiquity and translational medicine are effectively applied in some countries with more stable economies for other diseases and would help to prevent zoonotic pandemics such as COVID-19. On the other hand, the exchange of experiences and the adoption of professional containment activities such as those which are customary in veterinary practice, like isolation and quarantine measures, could be useful. The latter, unlike in human medicine, are instances when veterinary practices are widely and rigorously used because they represent the main principles for preventing the entrance and spread of diseases in naïve animal populations.

Surveillance measures represent the main strategy, considering these preventive needs. Active surveillance is important in the investigation of the potential pathogens of animals and the potentials of possible emergence in humans. This type of control would allow the acquisition of rich databases that would support specific and effective measures to control zoonotic epidemics. High-risk behaviors could also be identified, and health education activities could be initiated to change habits that contribute to and hinder the adaptation and dissemination of the pathogens.

Veterinarians are especially important in wildlife surveillance, which becomes a fundamental parameter in the control of emerging zoonoses because ecological changes, molecular variations of infectious agents, and wild animal-man interactions represent the main factors for the emergence of new pathogens. Therefore, the collaboration between veterinary communities linked to the monitoring of wildlife and human medical communities is crucial in the development of preventive strategies and must follow a double direction in the provision of early and specific information, which is not evident in most developing countries.

Microbiology studies combined with physiology, immunology, and behavioral ecology must be applied to asymptomatic animal hosts. They can demonstrate which mechanisms could explain the absence of clinical signs and can provide effective and applicable responses to humans. At the same time, they are specific animal models, which provide ideal conditions for the reproduction of the disease showing similarity to human responses and should be used to carry out vaccine and treatment tests before being applied to humans. Besides, prior knowledge of the etiologic agent, its intrinsic characteristics, and ecology can facilitate the faster development of vaccines and therapy drugs in cases of emergency and health risk.

Comparative medicine, in addition to *in vitro* analyzes, also covers field studies in the prevention of zoonosis. A good example of this is the monitoring of diseases that occur naturally in animal populations that can signal potential threats to human health. The use of sentinel animals, which have greater susceptibility, environmental exposure, or shorter life span has been very useful. The double meaning in interventions must be attributed to the One Health approach considering the risks shared between humans and animals.

Veterinary epidemiology allows alignment with disease forecasting and modeling studies through the application of georeferencing software that associates environmental variables, such as temperature, humidity, soil type, vector density, pathogen, host, exposure, and transit of animals and people. The convergence of factors that include the availability of these geocoded multi-temporal data and multi-professional collaborations worldwide would allow for the production of a sophisticated Geographic Information System under a holistic perspective for the development of research related to the control of zoonosis.

The molecular evolutionary aspects of zoonotic or potential pathogens must be constantly monitored. Genomic plasticity is a factor widely identified in viruses and bacteria within animal hosts and in hostile environmental conditions, such as thermal, oxidative, nutritional, and chemical stress, which could favor the selection pressure of more adapted, resistant, and/or pathogenic agents.

Several recent studies have highlighted the fundamental importance of the veterinarian's performance in the context of One Health. van Doremalen et al. (21) have demonstrated the preliminary efficacy of a vaccine tested on mice and *Rhesus* macaques against SARS-CoV-2 in partnership with a multi-professional team that includes veterinarians. In Chile, another multidisciplinary group, including veterinarians, developed an improved procedure to produce nanobodies using alpacas (*Lama pacos*) as donor species. The authors reported an optimized, fast, efficient, inexpensive, and simple density gradient method for nanobody selection and a sub-nanomolar affinity nanobody against the Spike receptor binding domain of SARS-CoV2. This proposed methodology may help in the generation of diagnostic and potentially therapeutic measures against COVID-19 and other infectious and emergent viruses (22). Sun et al. (23) reported a variant of H1N1 from pigs with the greatest pathogenic potential in humans. Their experimental work was carried out in veterinary laboratories in the USA. In India, Dhama et al. (24) have reported intense research performed with veterinarians in the development of vaccines and effective therapies against Ebola. Maki et al. (25) reported that the monitoring of wildlife allowed the implementation of control strategies such as the use of bait vaccines to control wild rabies.

Epidemics such as those already experienced and uncontrolled pandemics such as the current COVID-19 will continue to happen, not only because of the high capacity of zoonotic pathogens to carry out mutation, reassortment, and recombination processes that allow them to overcome barriers between species, geographical limitations, and adverse conditions but also due to the severe deficiency in the surveillance systems.

These factors coupled with few globalized and unified combat actions and the low interaction of the pillars of One Health focus, which involves the medical, veterinary and environmental communities, increases the risk of new emergencies in public health worldwide.

## Summary and Conclusion

Variations in behavior and different human activities, such as the consumption and sale of wild animals, the poor application of food security rules, the advance of urbanization into rural areas, and constant direct contact with animal reservoirs are recognized as the main risk factors that lead to outbreaks (26). All these factors can be tackled with preventive actions from a veterinary perspective, which are the resolutive competencies of this profession.

To avoid future emerging zoonoses, it is necessary to be prepared. The most effective way may be to maintain the natural barriers between animals that are reservoirs and human society, applying the conceptualization of the One Health doctrine in these actions. Despite being the weakest link, the veterinarian must assume a position of leadership in research and actions that primarily involve prevention and surveillance, which must be undertaken as an important part of maintaining public health, especially related to emerging and reemerging zoonoses.

## Author Contributions

RdM conceptualized the article and included the actions of the veterinary profession. DR included information about the current pandemic. GM contributed to writing and organization. HF provided adjustments to the One Health context. All authors approved the final version.

## Conflict of Interest

The authors declare that the research was conducted in the absence of any commercial or financial relationships that could be construed as a potential conflict of interest.
